# Supplementary material for: Benefit-cost analysis of coordinated strategies for control of rabies in Africa
Source: Nat Commun. 2023 Sep 7;14:5370. doi: 10.1038/s41467-023-41110-2 (PMC10484917; doi:10.1038/s41467-023-41110-2)
Supplement: Supplementary file 1 — Supplementary Information [file 41467_2023_41110_MOESM1_ESM.pdf]

Bucher et al. Benefit-cost analysis of coordinated strategies for control of rabies in Africa

Supplementary Information 0-3

Supplementary Information 0. Notations and descriptions

Notations

Supplementary Table 1. General subscripts used throughout the article.

Subscript	Definition
$-_c$	Variable or quantity defined for a particular country c.
$-_{\mathcal{C}}$	A vector of a particular variable or quantity defined for all countries in $\mathcal{C}$.
$-_Y$	Quantity evaluated for the year Y.

Supplementary Table 2. Distributions used for stochastic parameters.

Distribution	Definition
$\mathcal{N}(\mu, \sigma^2)$	Normal distribution with mean μ and standard deviation σ.
$\text{Beta}(\mu, \sigma, m, M)$	Beta distribution with mean μ, standard deviation σ, lower bound m and upper bound M. More details are in the “Beta distribution” section.

Supplementary Table 3. Notations used for the SEIR model for rabies in the dog population[1,2,3].

Symbol	Values	Description	Units
$N_{T=0,\text{dogs};c}$	$N_{T=0,\text{dogs};c} \sim \text{Beta}(\mu, \sigma, m, M)$	Total number of dogs in the country c in 2024 (start of the simulation).	Dogs
S	Cf. "Extrapolation of the transmission dynamics model"	Number of susceptible dogs.	Dogs
E	Cf. "Extrapolation of the transmission dynamics model"	Number of exposed dogs.	Dogs
I	Cf. "Extrapolation of the transmission dynamics model"	Number of infected dogs.	Dogs
V	Cf. "Extrapolation of the transmission dynamics model"	Number of vaccinated dogs.	Dogs
$N_{\text{rabid dogs}}$	Cf. "Extrapolation of the transmission dynamics model"	Cumulative number of ever-infected dogs.	Dogs

$\Delta N_{\text{rabid dogs};c,Y}$	Cf. "Extrapolation of the transmission dynamics model"	Difference between the cumulative numbers of ever-infected dogs from the year Y and the year $Y-1$ for the country c.	Dogs
$N_{\text{vaccinated dogs}}$	Cf. "Extrapolation of the transmission dynamics model"	Cumulative number of ever-vaccinated dogs.	Dogs
$\Delta N_{\text{vaccinated dogs}}$	Cf. "Extrapolation of the transmission dynamics model"	Difference between the cumulative numbers of ever-vaccinated dogs from the year Y and the year $Y-1$ for the country c.	Dogs
i_c	$i_c \sim \text{Beta}(\mu,\sigma,m,M)$	Annual increase of dogs in the country c.	Year^{-1}
β	1.0319227995	Transmission rate.	Week^{-1}
μ	0.0066	Dogs' birth/death rate.	Week^{-1}
σ	0.239	Rate of progression from exposed to infectious state.	Week^{-1}
δ	1.23	Disease induced death rate.	Week^{-1}
ν	0.95	Vaccination efficacy.	None
$\alpha(s_c)$	Cf. "Extrapolation of the transmission dynamics model"	Vaccination rate.	Week^{-1}
$\epsilon(s_c, s_{-c})$	Cf. "Extrapolation of the transmission dynamics model"	Reintroduction term (maintenance).	Dogs Week^{-1}
γ	$\gamma = \left(\frac{1}{52}\right) * \log(1+i_c)$	Weekly population growth rate.	Week^{-1}
$\min d(c, c')$	$\min d(c, c') \in [\![1,7]\!]$	Minimal number of countries between a vaccinated country c and an infected country c'.	Countries

Supplementary Table 4. Mathematical notations used for the payoff calculation and their description (Source Supplementary Data 4).

Symbol	Values	Description	Units
$P_{\text{PEP};c}$	$P_{\text{PEP};c} \sim \text{Beta}(\mu,\sigma,m,M)$	PEP price in $ in 2024 in the country c.	USD PEP^{-1}
$P_{\text{VAC};c,2024}$ $P_{\text{VAC};c,2025}$	$P_{\text{VAC};c,2024} \sim \text{Beta}(\mu,\sigma,m,M)$	Vaccination unit price in 2024 and 2025. For sensitivity analysis, the two prices were considered equal.	USD dog^{-1}

E_c	$E_c \sim \text{Beta}(2.3, 0.5, 1.5, 4)$	Exposure factor, number of annual bites per animal in the country c.	Bitten humans per dog
$\mathbb{P}_{\text{Clinical Case},c}$	$\mathbb{P}_{\text{Clinical Case},c} \sim \mathcal{N}(0.19, 0.01)$	Probability of developing a clinical case of rabies.	Clinical case per exposed human
$d_{\text{PEP},c}$	$d_{\text{PEP},c} \sim \mathcal{N}(1.5, 0.15)$	PEP demand factor due to the lack of knowledge about dogs' rabid statuses and to prevent all loss of human lives in the country c.	PEP per exposed human
$\mathbb{P}_{\text{PEP},c}$	$\mathbb{P}_{\text{PEP};c} \sim \text{Beta}(\mu, \sigma, m, M)$	Probability of receiving PEP in the country c.	PEP received (=humans saved) per clinical case
$GDP_{\frac{\text{forgone}}{\text{capita}},c}$	Cf. "Economic Valuation"	Foregone GDP per capita in the country c.	USD person^{-1}

Supplementary Table 5. Notation table for the game theoretical model.

Symbol	Values	Description
$\mathcal{G}$		A game in the game theoretic setting.
$c, \mathcal{C}$	$c \in \mathcal{C}$	A particular country c in the set of countries $\mathcal{C}$.
$s_c, \mathcal{S}_c$	$s_c \in \mathcal{S}_c$	A strategy s_c of the country c from the set of all possible strategies $\mathcal{S}_c$ of that country.
$\mathbf{s}_{-c}, \mathcal{S}_c$	$\mathbf{s}_{-c} \in \mathcal{S}_c$	A strategy profile $\mathbf{s}_{-c}$ composed from strategies of all countries excluding c. The set $\mathcal{S}_{-c}$ represents the set of all possible strategy profiles with all countries except the country c.
$\mathbf{s}_{\mathcal{C}}, \mathcal{S}_{\mathcal{C}}$	$\mathbf{s}_{\mathcal{C}} \in \mathcal{S}_{\mathcal{C}}$ $\mathcal{S}_{\mathcal{C}} = \mathcal{S}_{c_1} \times \ldots \times \mathcal{S}_{c_N}$	A strategy profile $\mathbf{s}_{\mathcal{C}}$ (vector of strategies for all countries) from the set of all possible strategies for all countries $\mathcal{S}_{\mathcal{C}}$.
$\mathbf{r}_{\mathcal{P}_c}, \boldsymbol{\mathcal{P}}_c$		A realization vector $\mathbf{r}_{\mathcal{P}}$ of a random vector of parameters $\boldsymbol{\mathcal{P}}_c$ for the country c.
$U_{\text{VAC};c}$	Cf. "Strategy Payoff Calculation"	Utility function for mass dog vaccination for the country c. It corresponds to the mass dog vaccination campaign cost in USD.
$U_{\text{PEP};c}$	Cf. "Strategy Payoff Calculation"	Utility function for PEP use in the country c. It corresponds to the cost (in USD) of PEP administration to humans exposed to rabies.
$U_{\text{HCE};c}$	Cf. "Strategy Payoff Calculation"	Utility function associated with the economic evaluation (in USD) of human lives lost due to rabies for the country c.

$U_{\text{HCEB};c}$	Cf. "Strategy Payoff Calculation"	Utility function associated with the economic evaluation (in USD) of all saved lives for the country c.
U_c	Cf. "Strategy Payoff Calculation"	Total utility function for the country c (in USD).
$U_c(s_c, \mathbf{s_{-c}}; \mathbf{r}_{\boldsymbol{\mathcal{P}}})$	Cf. "Strategy Payoff Calculation"	Evaluation of the total utility function for the country c given the strategy profile $(s_c, \mathbf{s_{-c}})$ and a realization vector $\mathbf{r}_{\boldsymbol{\mathcal{P}}}$ (in USD).
$\Delta U_c(s_c, \mathbf{s_{-c}}; \mathbf{r}_{\boldsymbol{\mathcal{P}}})$	Cf. "Strategy Payoff Calculation"	Payoff difference or evaluation of the difference between the utility function $U_c(s_c, \mathbf{s_{-c}}; \mathbf{r}_{\boldsymbol{\mathcal{P}}})$ and the baseline for the same realization vector $\mathbf{r}_{\boldsymbol{\mathcal{P}}}$ (in USD).

For each country, some parameters were considered as probability distributions to consider the uncertainty on these parameters. All these parameters are considered as a random vector $\forall c \in \mathcal{C},\ \boldsymbol{\mathcal{P}_c} = [N_{T=0,\text{dogs};c},\ i_c,\ P_{\text{PEP};c},\ P_{\text{VAC};c,2024},\ E_c,\ \mathbb{P}_{\text{Clinical Case},c},\ d_{\text{PEP},c},\ \mathbb{P}_{\text{PEP},c}]$.

Beta distribution

The random variable X is following a Beta distribution with shape parameters α and β if and only if its probability density function is

$$f_X(x) = \begin{cases} \frac{1}{B(\alpha,\beta)} x^{\alpha-1}(1-x)^{\beta-1} & \text{if } x \in [0,1] \\ 0 & \text{otherwise} \end{cases}$$

Where B is the Beta function.

We can transform the original Beta distribution, as defined before, in order to have a distribution with the following parameters: mean μ, standard deviation σ, lower bound of the support m and the upper bound of the support M.

$\text{Beta}(\mu, \sigma, m, M) = \text{Beta}(\alpha, \beta)$ with $\alpha = \left(\frac{\mu-m}{M-m}\right)\left(\frac{(M-\mu)(\mu-m)}{\sigma^2} - 1\right)$ and $\beta = \left(\frac{M-\mu}{\mu-m}\right)\alpha$

For the simulation, the lower bounds, means, and upper bounds for different parameters are in Supplementary Data 1. For each country, the standard deviation is calculated as follows:

**Parameter**	**Standard deviation calculation**
$N_{T=0,\text{dogs};c}$	$\sigma = 0.65 * \frac{M-m}{3.92}$
i_c	$\sigma = 0.6 * \frac{M-m}{3.92}$

$P_{\text{PEP};c}$	$\sigma = 0.6 * \frac{M-m}{3.92}$
$P_{\text{VAC};c,2024}$	$\sigma = 0.6 * \frac{M-m}{3.92}$
$\mathbb{P}_{\text{PEP},c}$	$\sigma = 0.06 * \frac{M-m}{3.92}$ for Algeria, Botswana, Gabon, Libya, Namibia, South Africa, Tunisia, Egypt, because the mean and the upper bound are too close to 1.<br>$\sigma = 0.25 * \frac{M-m}{3.92}$ for the rest.

Special log transformation

The following transformation is used on the payoff U_c to compare payoffs magnitudes and signs between countries in Supplementary figures 3 and 4:

$$u_{\log 10,c}(U_c) = \begin{cases} \log_{10} U_c\,, & \text{if } U_c > 1 \\ -\log_{10} -U_c, & \text{if } U_c < 1 \\ 0, & \text{otherwise} \end{cases}$$

Supplementary Information 1. Methods

Extrapolation of the transmission dynamics model

As a basis, the simplified deterministic ordinary differential equation for the homogenous model from Laager et al. for N'Djamena, Chad has been adapted[1]. This model allows pathogen reintroduction and, through a slight modification, allows for varying dog populations to derive the yearly country-specific dog-rabies cases incidence. The following mathematical formulation of the SEIR model was used for each country with country-specific values.

$$\begin{cases} \frac{dS}{dt} = \overbrace{\mu N_0 e^{\gamma t}}^{population\ growth} - \left(\overbrace{\nu\alpha}^{vaccination} + \overbrace{\mu}^{natural\ death}\right) S(t) - \overbrace{\frac{\beta S(t) I(t)}{S(t)+E(t)+I(t)+V(t)}}^{exposed} & \text{Suceptible} \\ \frac{dE}{dt} = \frac{\beta S(t) I(t)}{S(t)+E(t)+I(t)+V(t)} - \left(\overbrace{\sigma}^{infected} + \overbrace{\mu}^{natural\ death}\right) E(t) + \overbrace{\epsilon(s_c, s_{-c}, t) e^{\gamma t}}^{Reintroduction} & \text{Exposed} \\ \frac{dI}{dt} = \sigma E(t) - \left(\overbrace{\delta}^{disease\ induced\ death\ rate} + \overbrace{\mu}^{natural\ death}\right) I(t) & \text{Infected} \\ \frac{dV}{dt} = \nu\alpha(s_c, t) S(t) - \mu V(t) & \text{Vaccinated} \\ \frac{dN_{\text{rabid dogs}}}{dt} = \sigma E(t) & \text{Cumulated infected} \\ \frac{dN_{\text{vaccinated dogs}}}{dt} = \nu\alpha(s_c, t) S(t) & \text{Cumulated vaccinated} \end{cases}$$

The two last equations are used to capture the cumulated population of rabid dogs and of vaccinated dogs. In addition, to the standard terms of the model, we have two strategy-specific parameters. The vaccination rate $\alpha(s_c, t)$, which is different from zero during the vaccination campaign only. It is defined as follows:

$$\alpha(s_c, t) = \begin{cases} \begin{cases} 0.024, & \text{if } t \in [0, 104] \\ 0, & \text{otherwise} \end{cases} & \text{if } s_c = vac \\ 0, & \text{if } s_c = pep \end{cases}$$

The second strategy-dependent term is the reintroduction term $\epsilon(s_c, s_{-c}, t)$, that is equal to zero while the vaccination is effective and the pathogen is not reintroduced. We simplify by assuming that the pathogen takes 39 weeks to go through a country to infect a neighbouring one. Mathematically, we define it as follows:

$$\epsilon(s_c, s_{-c}, t) = \begin{cases} \begin{cases} \epsilon_0, & \text{if } t \in [104 + 39 * \min d(c, c');\ +\infty[\text{ and } \exists c' \in \mathcal{C} | s_{c'} = pep \\ 0, & \text{otherwise} \end{cases} & \text{if } s_c = vac \\ \epsilon_0, & \text{if } s_c = pep, \forall t \in [0, \infty[\end{cases}$$

The input parameters for the SEIR model are derived from recent publications in the same context[1, 2, 3]. These input parameters are presented in Supplementary table 3.

The number of dogs per country has been derived from utilizing the dog: human ratio for Africa as published by Knobel et al. with a distinction between urban and rural populations[4]. The human population distinguished by rural and urban was derived from the World Bank and the total population as well as the population growth rate by the United Nations World Population Prospects 2019[5].

The underlying assumption is that, on average, a homogeneous endemic dog-rabies cases incidence of 1.9 per 100'000 dogs per week over all 48 countries. Pairing these results with the 2013 animal bite survey on animal bite injuries, a multiplication by 2.3 in the same geographical location[6] results in the corresponding number of humans exposed to rabid dogs. As 19% of all bite victims from rabid dogs develop clinical rabies that results in death[7], an estimation of all countries' absolute number of human lives lost per year under no intervention can be conducted.

Possible intervention strategies under investigation

Currently, canine rabies human lives lost are being prevented through the administration of post-exposure prophylaxis (PEP). Recent studies have shown that rabies transmission from dogs to humans can be interrupted through a dog vaccination campaign[8], but that pathogen reintroduction occurs outside the vaccination area[1]. Considering these preconditions, we investigate the following three possible scenarios.

i. Treatment of dog bite victims with PEP
ii. Treatment of dog bite victims with PEP and a dog vaccination campaign with pathogen reintroduction
iii. Treatment of dog bite victims with PEP and a dog vaccination campaign with interrupted pathogen reintroduction can be achieved through country coordination.

The first scenario can be seen as a scenario where there is no collaboration between the veterinary and public health authorities which we refer to as the baseline strategy. In contrast, the latter two follow a One-health approach, aiming at an incremental benefit of closer cooperation between human and animal health and other sectors[9]. The SEIR model was implemented in R version 4.1.2 and in Python 3.9.5. In Python solved with the function odeint from scipy library (version 1.10.1), this function calls FORTRAN77 library ODEPACK and

precisely LSODE solver (Livermore Solver for Ordinary Differential Equations)[10]. The post-treatment was executed with Python 3.8. Moreover, all source code is functioning with this Python version.

Economic Valuation

To calculate the payoffs of the strategies, the intervention costs (administered PEP and vaccination campaign costs) constitute the cost factors. The averted monetized years of life lost (YLL) constitute the strategy benefit. Regarding a limited PEP reach and compliance, rabies-induced monetized YLL due to limited PEP reach and compliance constitute an additional cost factor.

As an overall assumption, all monetized effects are subject to a universal 3% GDP growth rate and a yearly 5% discount rate over all countries in scope.

The economic valuation of the vaccination campaign follows the public costs approach from a previous study in N'Djamena, which divides the total costs into material/logistics and awareness campaign costs[2, 11]. The material costs are assumed to have stayed constant since 2016, whereas the logistic and awareness campaign costs have been subject to changes in the GDP. The former two were then normed to the 48 countries' specific GDP. GDP values for 2024 are obtained from the latest available value in the World Bank database on GDP per capita in current US$[12] except for Eritrea and South Sudan, where the values were unavailable and attained from the International Monetary Fund[13] and then perpetuated with a 3% annual growth rate.

The PEP costs are estimated at a uniform cost of 125 USD per treatment, including rabies immune globulin (RIG), with an expected price of 125 USD (from the co-author's (Jakob Zinsstag) expert's opinion) with a maximum price of 150 USD and a minimum value of 100 USD (95% CI: 106.5 USD – 143.5 USD) for the base year 2024. In most African countries, PEP is not regularly available. Most often PEP can be found in larger cities but not in rural areas. Most rabies exposed people die because of the lack of availability of PEP and the lack of compliance with repeated PEP vaccination[14]. The probability of receiving a timely PEP treatment is estimated derived from the 2015 Hampson et al. study[15].

As healthcare providers have most often no information on whether a suspected rabid dog is truly rabid, the number of exposed humans must be multiplied by a factor of 1.5 to prevent all human lives from being lost, as approximately 2/3 of human suspected rabies exposure

cases are actually at risk of developing clinical rabies[16, 17]. This follows from the logic, that our SEIR model calculates the number of humans that have truly been bitten by a rabid dog.

The bite victims' age distribution is considered for the YLL's economic valuation. 19% of the bite victims are aged 0-4, 36% are aged 5-15, and 45% are above 15 years[18]. The average age within these age groups for every country was calculated using the World Population Prospects 2019 database for 2020. The life expectancy (LEB) at an exact age was derived from the same database. Changes in the LEB as of 2024 were not considered[5].

The assessment of the GDP contribution was done following the OECD definition of the working-age population, which defines the age interval between 15 to 64[19] as the working age. The combination of the years of life lost with GDP contribution derived from the age distribution with the country-specific GDP contribution results in the weighted average YLL with GDP contribution for one average bite victim in a specific country.

The countries' GDP per capita was then utilized as a proxy for the economic contribution of an individual in a given year[20]; these values were obtained from the World Bank database on GDP per capita in current US$[12]. For Eritrea and South Sudan, these values were attained from the International Monetary Fund database[13].

As 19% of exposed humans develop clinical rabies [7], the yearly human lives lost can be directly derived from the exposed humans. The combination of the absolute number of "yearly human lives lost" with the expected years in the working-age (15 to 64) from an average bite victim.

Strategy Payoff Calculation

On the one hand, the results of our SEIR model provide us with estimations of the vaccinated dog population and rabid dog population. The first one is used to calculate the cost of the vaccination campaign. The second is used to estimate the cost of PEP administration and the monetized YLL. Using the notations from Supplementary Information 0, we explicit different costs for each country $c \in \mathcal{C}$.

i. Mass dog vaccination cost:

$$U_{\mathrm{VAC};c}(s_c, \mathbf{s}_{-c}) = -\sum_{Y=2024}^{Y=2054} \Delta N_{\text{vaccinated dogs};c,Y} * P_{\mathrm{VAC};c,Y}$$

ii. PEP administration cost:

$$U_{\text{PEP};c}(s_c, \mathbf{s}_{-c}) = -\sum_{Y=2024}^{Y=2054} P_{\text{PEP};c,Y} * d_{\text{PEP}} * \overbrace{E * \Delta N_{\text{rabid dogs};c,Y}}^{Exposed\ humans}$$

iii. Monetized Years of Life Lost (YLL) or Human Capital Effect (HCE) cost:

$$U_{\text{HCE};c}(s_c, \mathbf{s}_{-c}) = -\sum_{Y=2024}^{Y=2054} (1 - \mathbb{P}_{\text{PEP}}) * \overbrace{\mathbb{P}_{\text{Clinical Case}} * E_c * \Delta N_{\text{rabid dogs};c,Y}}^{Number\ of\ clinical\ cases} * GDP_{\frac{\text{forgone}}{\text{capita}};c,Y}$$

iv. Human Capital Effect benefit:

$$U_{\text{HCEB};c}(s_c, \mathbf{s}_{-c}) = +\sum_{Y=2024}^{Y=2054} \overbrace{\mathbb{P}_{\text{Clinical Case}} * E_c * \Delta N_{\text{rabid dogs};c,Y}}^{Number\ of\ clinical\ cases} * GDP_{\frac{\text{forgone}}{\text{capita}};c,Y}$$

The last term would simplify during the comparison as the human capital effect benefits would be the same in different scenarios. Therefore, we will not include it in the total payoff. Finally, the total payoff of a country c is:

$$U_c(s_c, \mathbf{s}_{-c}) = U_{\text{VAC};c}(s_c, \mathbf{s}_{-c}) + U_{\text{PEP};c}(s_c, \mathbf{s}_{-c}) + U_{\text{HCE};c}(s_c, \mathbf{s}_{-c})$$

We also define the gains or losses of the strategy profile $\mathbf{s}_{\mathcal{C}}$ compared to the baseline strategy (PEP administration by all countries), given a realization $\mathbf{r}_{\mathcal{P}_c}$ of a set of stochastic parameters $\boldsymbol{\mathcal{P}}_c$ for a country c.

$$\Delta U_c(\mathbf{s}_{\mathcal{C}}; \mathbf{r}_{\mathcal{P}}) = U_c(\mathbf{s}_{\mathcal{C}}; \mathbf{r}_{\mathcal{P}}) - U_c(\mathbf{s}_{\mathcal{C};\text{PEP}}; \mathbf{r}_{\mathcal{P}})$$

This way, we can perform a sensitivity analysis and compare different strategy profiles, always using the same realization for the strategy and the baseline. Because the realization is independent of the strategy, we can't compare a strategy profile and the baseline with different realizations.

Sensitivity analysis and consideration of uncertainty

For including the uncertainty in our modelling, we considered for each country the following parameters as distributions: the initial dog population, the annual increase in the dog population, the unit price of vaccination, the unit price of post-exposure prophylaxis (PEP), the exposure factor (2.3 in the literature[6] used as a mean), the probability of developing clinical symptoms (0.19 in the literature[7] used as a mean), the probability of receiving PEP and demand of PEP. The used distributions are described in Supplementary Information 0, and the used data for the bounds are in the Supplementary Data 1.

Using these distributions as input for the parameters for each country, we estimated the confidence intervals and distributions of outputs of interests, either using Monte Carlo

simulations with Latin hypercube sampling or Monte Carlo simulations during the Sobol experiment. The following Supplementary table summarizes different outputs and methods used.

Variable	Computed output of interest	Input Stochastic parameters	Method
**Total dog population (N_T)**	Confidence interval	• $N_{T=0,\text{dogs};c}$<br>• i_c	Monte Carlo with Latin hypercube sampling (200000 per country)
**Rabid dog population ($N_{\text{rabid dogs};c,Y}$)**	Confidence interval<br>Distribution	• $N_{T=0,\text{dogs};c}$<br>• i_c	Monte Carlo with Latin hypercube sampling (200000 per country)
**Exposed humans ($E_c * N_{\text{rabid dogs};c,Y}$)**	Confidence interval<br>Distribution	• $N_{T=0,\text{dogs};c}$<br>• i_c<br>• E_c	Monte Carlo with Latin hypercube sampling (200000 per country)
**Clinical cases estimation ($\mathbb{P}_{\text{Clinical Case},c} * E_c * N_{\text{rabid dogs};c,Y}$)**	Confidence interval<br>Distribution	• $N_{T=0,\text{dogs};c}$<br>• i_c<br>• E_c<br>• $\mathbb{P}_{\text{Clinical Case},c}$	Monte Carlo with Latin hypercube sampling (200000 per country)
**Number of lives lost**	Confidence interval<br>Distribution	• $N_{T=0,\text{dogs};c}$<br>• i_c<br>• E_c<br>• $\mathbb{P}_{\text{Clinical Case},c}$<br>• $\mathbb{P}_{\text{PEP},c}$	Monte Carlo using the results of "Clinical cases estimation" and the distribution of $\mathbb{P}_{\text{PEP}}$ (1000000 per country)
**Country payoff (U_c)**<br>**Country gains (ΔU_c)**	Confidence interval<br>Distribution	• $N_{T=0,\text{dogs};c}$<br>• i_c<br>• E_c<br>• $\mathbb{P}_{\text{Clinical Case},c}$<br>• $P_{\text{PEP};c}$<br>• $P_{\text{VAC};c,2024}$<br>• $d_{\text{PEP},c}$<br>• $\mathbb{P}_{\text{PEP},c}$	Monte Carlo during evaluations for Saltelli sensitivity algorithm (500000 per country)
**Country gains (ΔU_c)**	Sobol Indices		Saltelli sensitivity algorithm with appropriate sampling (500000 per country)

To investigate the impact of the input parameters on the result, we conducted a variance-based sensitivity analysis, calculating the first-order and total-order Sobol indices[21] for the gains and losses of the strategy profile $\boldsymbol{s_{C;VAC}}$, where all countries mass vaccinate dogs, and the strategy profile $\left(s_{c;\text{VAC}}, \mathbf{s_{-c;PEP}}\right)$, where only the studied country c vaccinates the dogs. The Sobol indices were computed using the Saltelli sensitivity algorithm[22] implemented in the Python library Openturns (version 1.21) and computed on the computing cluster scicore of the University of Basel. The computed Sobol indices are presented as heatmaps in the following Supplementary figures (Supplementary fig. 1 and fig. 2) and can be retrieved in the Supplementary Data 3.

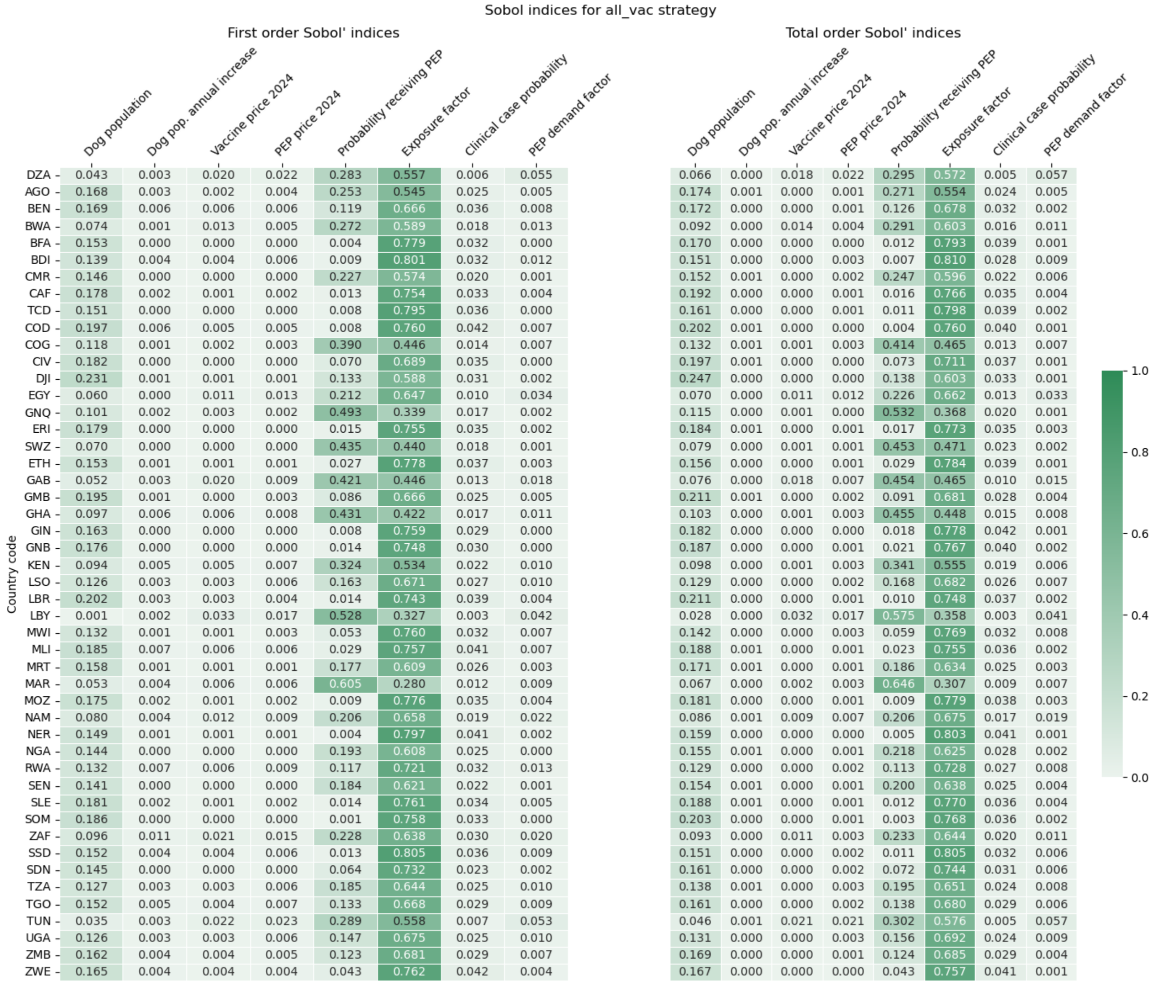

Supplementary Figure 1. Sobol indices for the cooperative strategy, all countries vaccinate dogs. ***a*** *First-order Sobol indices.* ***b*** *Total-order Sobol indices.*

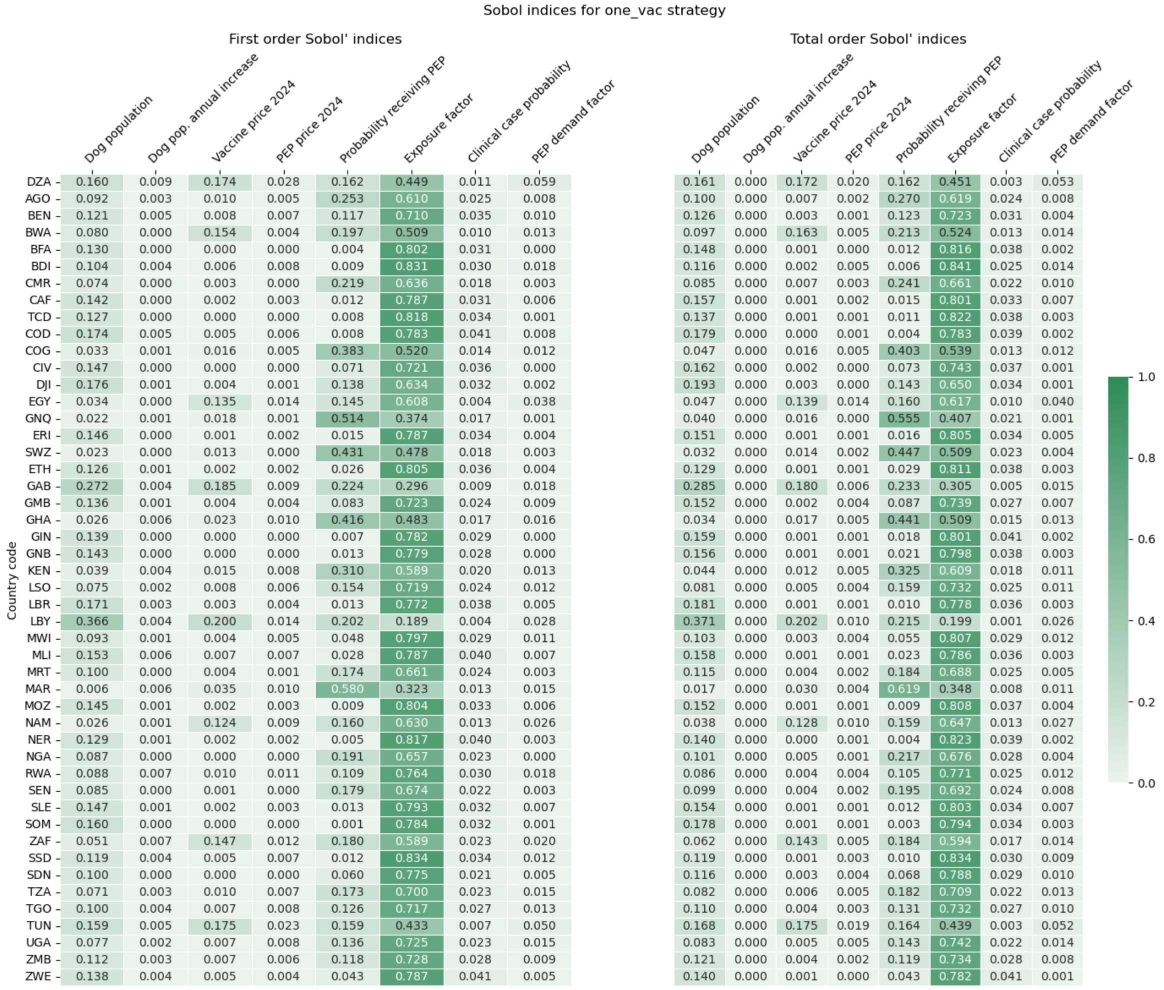

Supplementary Figure 2. Sobol indices for the strategy profile where only the selected country vaccinates dogs. ***a*** *First-order Sobol indices.* ***b*** *Total-order Sobol indices.*

On average, we have similar values for Sobol indices in the two scenarios, "complete vaccination" and "vaccination with reintroduction". The most important factor is the exposure factor with a total order Sobol indice's mean value of 0.66 ("all_vac") and 0.68 ("one_vac"). Then, we have the probability of receiving PEP, 0.17 ("all_vac") and 0.15 ("one_vac"). The third noticeable factor is the initial dog population with total Sobol indices of 0.15 ("all_vac") and 0.12 ("one_vac"). We can also observe that our first-order and total-order Sobol indices are relatively close. The average of the absolute difference between the first- and total-order indices is about 0.003 for both scenarios. It means that the interactions between our variables are negligible.

We observe that the vaccination price played a more significant role in the model's output with reintroduction for the countries where we used the reduced variance for the probability of receiving PEP. Instead of values close to 0, we find around 0.16. As the probability of

receiving PEP is closer to 1, the HCE term is becoming closer to 0. Therefore, the model is almost reduced to the payoff without HCE, so the vaccination price becomes a more important factor.

Mapping the Game

The last part of the results section utilizes the strategy payoffs to illustrate the overarching game and enable discussion of the strategic situation.

Supplementary Information 2: Modelling of the Game "Coordinated dog rabies mass vaccination against incomplete PEP"

Game description

Let's consider the strategic form of the "Coordinated dog rabies mass vaccination against incomplete PEP" $\mathcal{G} = (\mathcal{C}, \mathcal{S}, \{U_c\}_{c\in\mathcal{C}})$, where $\mathcal{C}$ is the players' set and is constituted of all African countries, the cardinality of $\mathcal{C}$ is 48. $\{U_c\}_{c\in\mathcal{C}}$ is the set of utilities, as defined in the previous section, even if, in practice, we will be interested more in the gains ΔU_c. $\mathcal{S}$ is the set of strategies, each country has a choice between two strategies: (i) mass dog vaccination (vac) and (ii) do nothing (pep), meaning that we react to human cases with PEP administration. We define a particular strategy of a country $c \in \mathcal{C}, s_c \in \mathcal{S}_c = \{\text{vac, pep}\}$, and the vector $\mathbf{s}_{-c} \in \mathcal{S}_{-c} = \{\text{vac}, \text{pep}\}^{47}$ as a strategy profile of other players excluding the player c. A strategy profile for the game $\mathcal{G}$ is a vector of strategies considering all players, $\mathbf{s}_{\mathcal{C}} : \mathcal{C} \to \mathcal{S} = \{\text{vac}, \text{pep}\}^{48}$. We also define some particular strategy profiles:

- $\mathbf{s}_{\mathcal{C};\mathbf{VAC}} = (\text{vac})^{48}$, all countries mass vaccinate dogs.
- $\mathbf{s}_{\mathcal{C};\mathbf{PEP}} = (\text{pep})^{48}$, no one mass vaccinates dogs. This is the baseline scenario.
- $\mathbf{s}_{\mathcal{C};\boldsymbol{n}} = \sigma((\text{vac})^{n}, (\text{pep})^{48-n})$, n countries vaccinate dogs, whereas $48 - n$ don't. In this context $\sigma(.)$ defines a permutation.

Game theory definitions

We recall some important definitions adapted to our notation.

Def. 1 (Best response)

The best response (BR) of player c to the (reduced) strategy profile s_{-c} is the correspondence given by:

$$\text{BR}_c(s_{-c}) = \arg\max_{s_c \in \mathcal{S}_c} U_c(s_c, \mathbf{s}_{-c})$$

Def. 2 (Nash equilibrium)

Considering the game $\mathcal{G} = (\mathcal{C}, \mathcal{S}, \{u_c\}_{c\in\mathcal{C}})$, the vector s^* is a Nash equilibrium if:

$$\forall c \in \mathcal{C}, \forall s_c \in \mathcal{S}_c, u_c(s_c^*, \mathbf{s}_{-c}^*) \geq u_c(s_c, \mathbf{s}_{-c}^*)$$

Def. 3 (Dominant Strategy):

Considering the strategic form game $\mathcal{G} = (\mathcal{C}, \mathcal{S}, \{U_c\}_{c\in\mathcal{C}})$. The strategy s_c^D is a dominant strategy for player c if:

$$\forall \mathbf{s}_{-c} \in \mathcal{S}_{-c}, \forall s_c \in \mathcal{S}_c, u_c(s_c^D, \mathbf{s}_{-c}) \geq u_c(s_c, \mathbf{s}_{-c})$$

It is strictly dominant if the inequality is strict. By definition, a rational player is maximizing his utility using all available information[23, 24]. As a consequence, a rational player will always play a dominant strategy if it exists.

Def. 4 (Pareto-optimal profile)

A strategy profile $\mathbf{s}_{\mathcal{C}}^{\mathbf{PO}}$ is a Pareto-optimal if and only if $\forall c \in \mathcal{C}, \forall \mathbf{s}_{\mathcal{C}} \in \mathcal{S}_{\mathcal{C}}, u_c\left(\mathbf{s}_{\mathcal{C}}^{\mathbf{PO}}\right) \geq u_c(\mathbf{s}_{\mathcal{C}})$.

Game analysis: non-cooperative setting

For the first analysis, let's consider the average realization of our stochastic parameters $\mathcal{P}$, using the mean values, we calculate or estimate the countries' payoffs based on the strategy profile. In the following Supplementary table, we summarize for each studied strategy profile the details of the estimation.

Supplementary Table 6. Methodology for calculation of different values for strategy analysis.

Strategy profile	Description	Number of possible combinations	Method	Sample size for the experiment
$\mathbf{s}_{\mathcal{C};\mathbf{PEP}}$	Baseline, no one vaccinate	1	Direct calculation	1
$\mathbf{s}_{\mathcal{C};\mathbf{VAC}}$	All countries mass vaccinate	1	Direct calculation	1
$\mathbf{s}_{\mathcal{C};\mathbf{Nash}}$	Nash equilibrium profile	1	Direct calculation	1
$\mathbf{s}_{\mathcal{C};\mathbf{1}}$	Only one country out of 48 vaccinates	48	Direct calculations for all 48 possibilities. Except for the strategy profile, where the studied country is mass vaccinating, its payoff difference equals zero, so we report the non-zero value for each country.	48
$\mathbf{s}_{\mathcal{C};\mathbf{47}}$	Only one country out of 48 doesn't vaccinate	48	Average of direct calculations for all 48 possibilities	48

$\mathbf{s}_{\mathcal{C};\mathbf{12}}$	12 countries vaccinate, while the others don't	$\approx 7 * 10^{10}$	Average of a sample of 271 simulations, with random vaccinated countries	271 (CI=90%, E=5%)
$\mathbf{s}_{\mathcal{C};\mathbf{36}}$	36 countries vaccinate, while the others don't	$\approx 7 * 10^{10}$	Average of a sample of 271 simulations, with random vaccinated countries	271 (CI=90%, E=5%)
$\mathbf{s}_{\mathcal{C};\mathbf{24}}$	Half vaccinate, half don't	$\approx 3.2 * 10^{13}$	Average of a sample of 271 simulations, with random vaccinated countries	271 (CI=90%, E=5%)

The results are available in the additional content "Supplementary Data 2". In the mean case, we find that all countries obtain the highest payoff if they all vaccinate. It would be the Pareto-optimal strategy. For all countries except Botswana, Algeria, Egypt, Gabon, Libya, Namibia, Tunisia, and South Africa, the payoff of the vaccination strategy is higher than the baseline, even if they are alone to vaccinate. That means that mass dog vaccination is the dominant strategy for those countries. For the countries cited above, there is no dominant strategy because their payoff is higher if everybody cooperates, but if there is at least one country to defect, there are no more advantages for them to cooperate. In the non-cooperative setting, we can see that the best response strategy for them is to defect. Let $\mathcal{C}_{\text{PEP}} = \{\text{Botswana}, \text{Algeria}, \text{Egypt}, \text{Gabon}, \text{Libya}, \text{Namibia}, \text{Tunisia}, \text{South Africa}\}$ the set of countries with no dominant strategy, thus, $\mathcal{C}_{\text{VAC}} = \mathcal{C} \backslash \mathcal{C}_{\text{PEP}}$ the set of countries with mass dog vaccination as the dominant strategy. Therefore, we have the following profile as a Nash equilibrium: $\mathbf{s}_{\mathcal{C},\mathbf{Nash}} = \left(\mathbf{s}_{\mathcal{C}_{\mathbf{PEP}}} = \text{pep}, \mathbf{s}_{\mathcal{C}_{\mathbf{VAC}}} = \text{vac}\right)$.

We summarize their payoffs in the following Supplementary figures for the cooperative strategy $s_{\mathcal{C};\text{VAC}}$ and in the strategies profile, where they are the only ones to vaccinate $\forall c \in \mathcal{C}$, $\left(s_{c;\text{VAC};}\mathbf{s}_{-\boldsymbol{c};\mathbf{PEP}}\right)$.

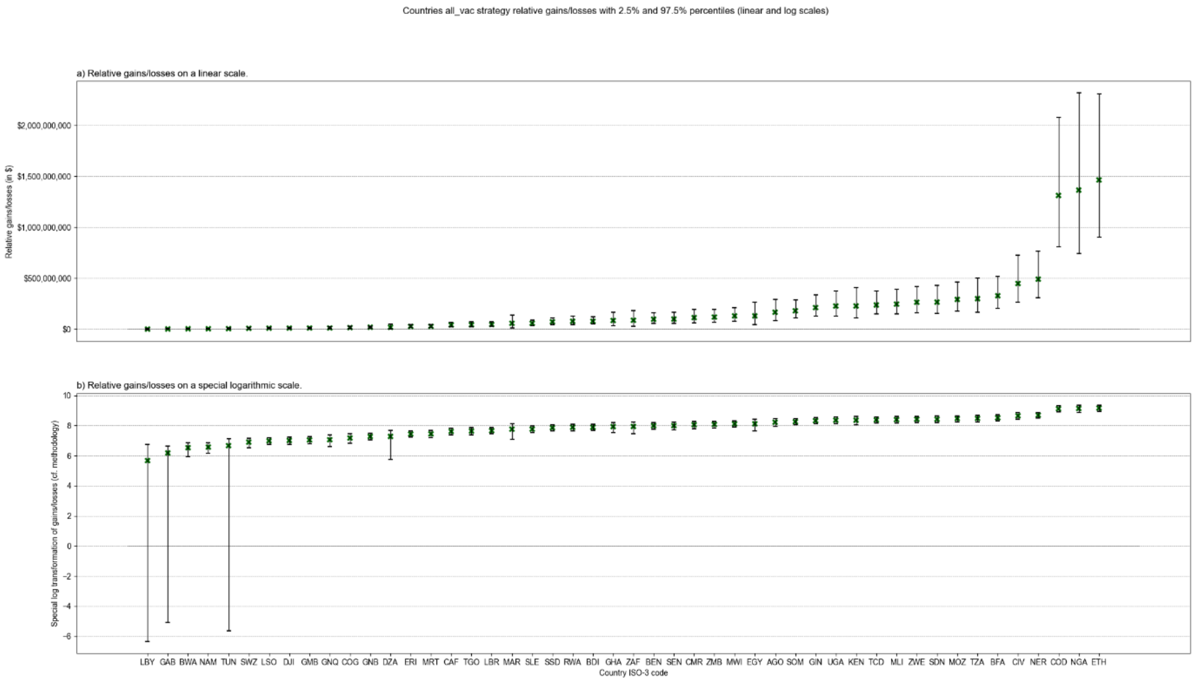

Supplementary Figure 3. Payoff difference (compared to the baseline) with 95% confidence interval per country in the cooperative scenario. The x marker represents the mean value of the empirical distribution found from the Monte-Carlo experiment during the sensitivity analysis, the marker is red if the value is negative and green if the value is positive. This value also corresponds to the deterministic case. Error bars represent the 95% confidence interval with the lower limit of 2.5 percentile and upper limit of 97.5 percentile of the empirical distribution. For each country, n=500000 independent samples from the Monte-Carlo experiment were used. ***a*** *The payoff in USD.* ***b*** *The payoff is transformed with a log transformation as defined in Supplementary Information 0.*

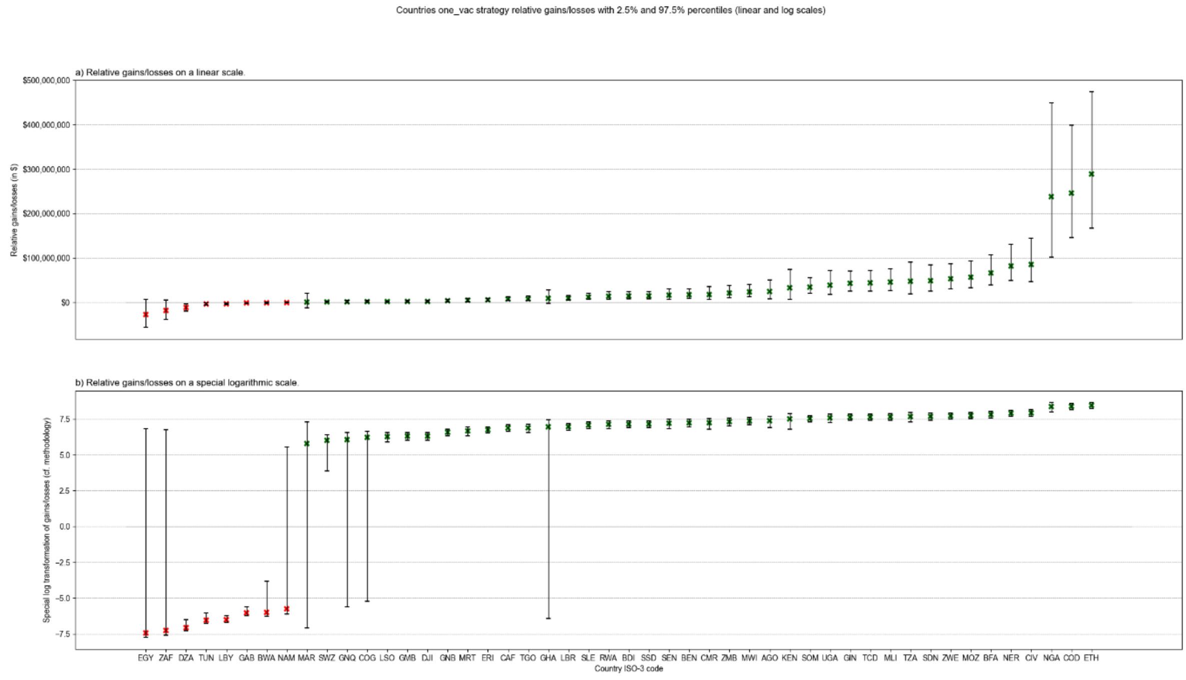

Supplementary Figure 4. Payoff difference (compared to the baseline) with 95% confidence interval per country in the scenario where only the selected country mass vaccinates dogs. It is a summary of 48 strategy profiles, with each country using a "One VAC" strategy. The x marker represents the mean value of the empirical distribution found from the Monte-Carlo experiment during the sensitivity analysis, the marker is red if the value is negative and green if the value is positive. This value also

corresponds to the deterministic case. The vertical line represents the 95% confidence interval with the lower limit of 2.5 percentile and upper limit of 97.5 percentile of the empirical distribution. ***a*** *The payoff in USD.* ***b*** *The payoff is transformed with a log transformation as defined in Supplementary Information 0.*

Breakeven points

We calculated the breakeven points using the yearly payoffs per country to compare different strategies. We compare 5 strategy profiles: (i) the baseline strategy with only PEP use, (ii) the Pareto-optimal solution, i.e. full vaccination profile, (iii) strategy profile where only one country does not mass vaccinate dogs, (iv) half of countries mass vaccinate dogs, (v) Nash equilibrium profile. In Supplementary Figure 5, we can see the cumulated payoffs without HCE for different strategies over the years. By excluding the HCE cost, we compare only the cost of vaccination and of PEP use. The first result we can observe is that over all 48 countries, we have a breakeven point between the baseline strategy profile and the full vaccination strategy profile in 2033. The second is that the payoff (without HCE) of the baseline strategy profile is always greater than the payoffs of strategy profiles (iii), (iv) and (v). It is explained by the fact that we invest in mass dog vaccination in those profiles. However, because of the reintroduction, we reach the endemic equilibrium and thus rely once again on the PEP in the same proportions as in the baseline profile. In Supplementary Figure 6, we consider aggregated total payoffs (with HCE) for the same strategy profiles. We can observe a significant shift in the results. All strategy profiles are more profitable after the first year than the baseline profile. We can also add that the payoffs of Nash equilibrium profile (v) and of the strategy profile (iii) are pretty close, meaning that the countries from $\mathcal{C}_{\mathrm{PEP}}$ are not gaining much by cooperating if there is at least one defector.

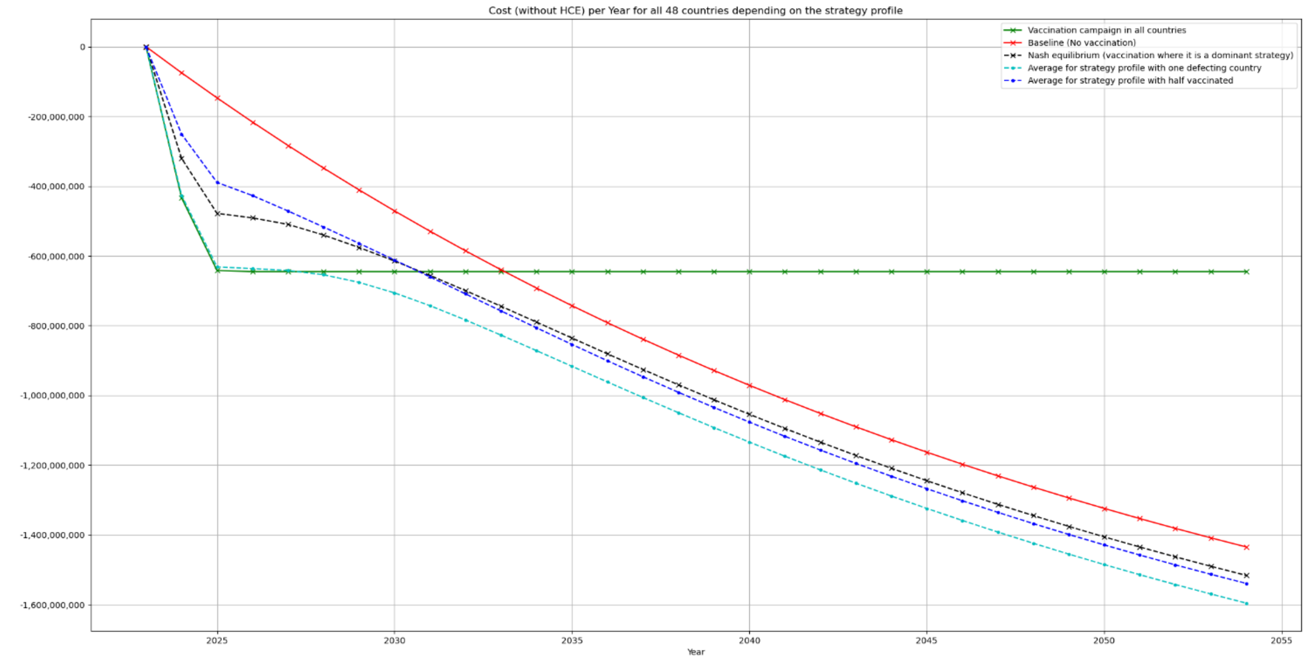

Supplementary Figure 5. Cumulated payoffs without HCE aggregated over all 48 countries by year for particular strategy profiles. Baseline strategy (i) in red, full cooperation (ii) in green, one defecting (iii) in cyan, half mass vaccinate (iv) in blue, Nash equilibrium (v) in black.

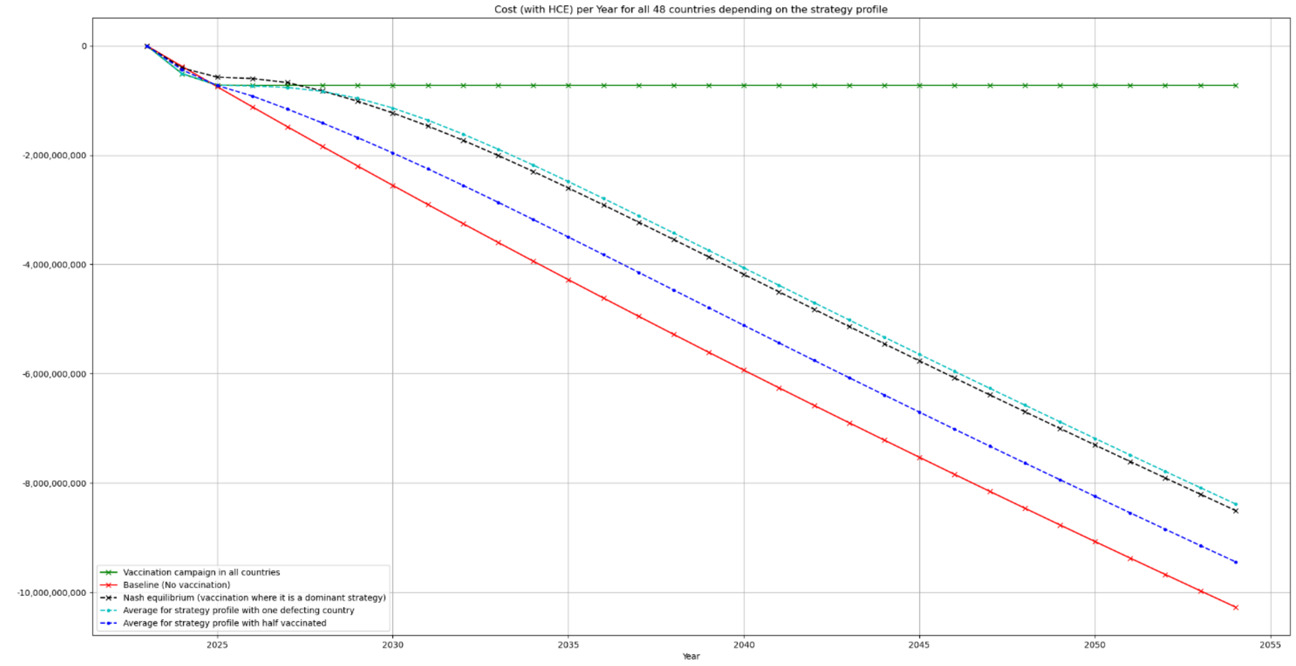

Supplementary Figure 6. Cumulated payoffs (including HCE) over all 48 countries by year for particular strategy profiles. Baseline strategy (i) in red, full cooperation (ii) in green, one defecting (iii) in cyan, half mass vaccinate (iv) in blue, Nash equilibrium (v) in black.

On Supplementary figures 7 and 8, we have a detailed view of the difference between the PEP (baseline) strategy profile and the cross-country vaccination program costs. On the Supplementary figure 7, we exclude the human capital effect cost, whereas we include it on

the Supplementary figure 8. We can see that without HCE, some countries will never meet their breakeven point, meaning that even if everybody mass vaccinate dogs, the initial vaccination program cost is greater than the continuous purchasing of post-exposure prophylaxis. The situation changes when we include the human capital effect cost, all countries benefit from the coordinated dog vaccination program. Moreover, for 18 countries (38%), it is profitable since the beginning of the dog rabies vaccination program, while after 4 years, 39 countries (81%) benefit from the program.

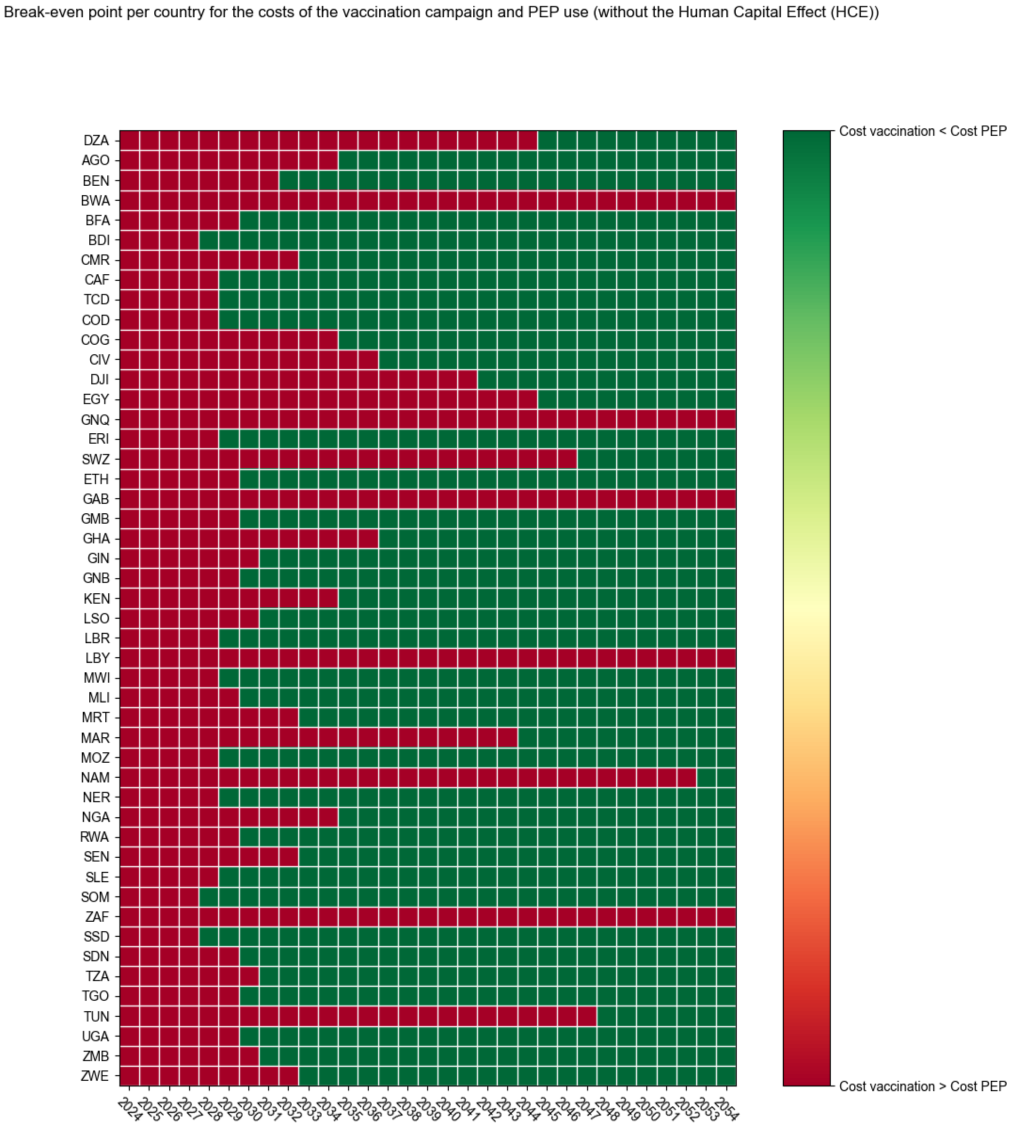

Supplementary Figure 7. Breakeven points for the vaccination campaign and the PEP for the Pareto-optimal solution excluding the human capital effect. For some countries, the regional vaccination campaign is never profitable during the studied period.

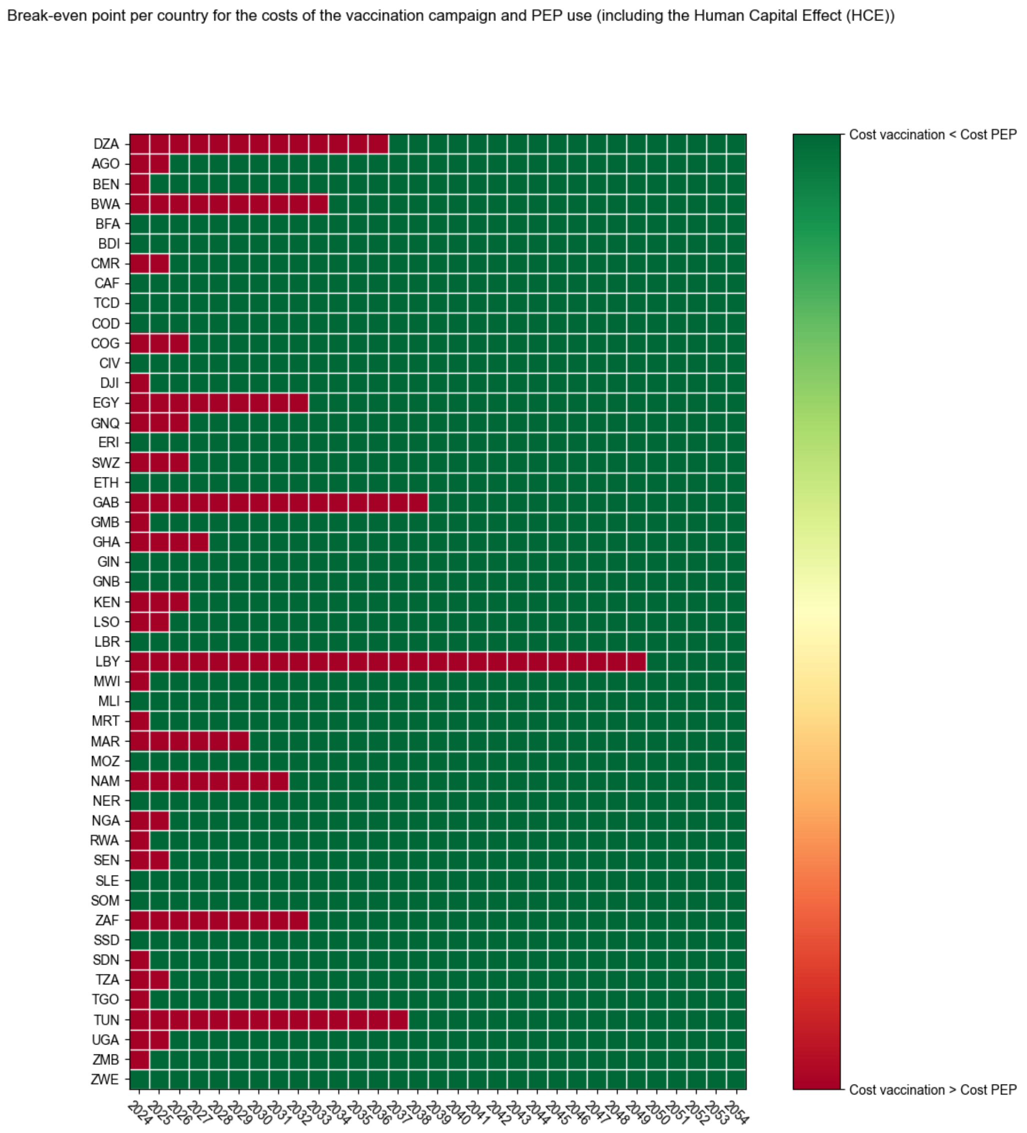

Supplementary Figure 8. Breakeven points for the vaccination campaign and the PEP for the Pareto-optimal solution including the human capital effect. For most countries, the regional vaccination campaign is profitable from the end of the second year.

Game analysis: cooperative setting

In the cooperative setting, we can take the two coalitions $\mathcal{C}_{\text{VAC}}$ and $\mathcal{C}_{\text{PEP}}$ as defined before and simplify the game as a two-person game with the gains compared to the baseline as the payoff. Based on the Monte Carlo simulations, we have the following payoff matrix (Supplementary table 7) in billions USD with a 95% confidence interval. For the coalition $\mathcal{C}_{\text{VAC}}$, the payoffs are $(0,0)$ for the baseline strategy, whereas $(1.7, 9.3)$ for the vaccination strategy. Therefore, $\forall s_{\mathcal{C}_{\text{PEP}}} \in \mathcal{S}_{\mathcal{C}_{\text{PEP}}}, U\left(\text{vac}_{\mathcal{C}_{\text{VAC}}}, s_{\mathcal{C}_{\text{PEP}}}\right) > U\left(\text{pep}_{\mathcal{C}_{\text{VAC}}}, s_{\mathcal{C}_{\text{PEP}}}\right)$. So, the vaccination strategy is a strictly dominant strategy for the coalition $\mathcal{C}_{VAC}$. We highlighted in bold the dominant strategy of the coalition $\mathcal{C}_{\text{VAC}}$ in the payoff matrix.

Supplementary Table 7. Payoff matrix with coalitions. We have the payoff of the coalition $\mathcal{C}_{VAC}$ on the left side of the tuple and the payoff of the coalition $\mathcal{C}_{PEP}$ on the right side. In bold, we highlighted the dominant strategy of the coalition $\mathcal{C}_{VAC}$ and the associated payoffs.

Coalitions		$\mathcal{C}_{\text{PEP}}$	
	Strategies	pep	vac
$\mathcal{C}_{\text{VAC}}$	pep	$(0, \quad 0)$	$(0, \quad -0.066\ [-0.1; -0.025])$
	**vac**	$(\mathbf{1.7\ [1.4; 2.1]}, \quad \mathbf{0})$	$(\mathbf{9.3\ [7.7; 11.2]}, \quad \mathbf{0.25\ [0.13; 0.41]})$

As a rational player, the coalition $\mathcal{C}_{\text{VAC}}$ will play its dominant strategy, i.e. mass dog vaccination. If the coalition $\mathcal{C}_{\text{PEP}}$ is coordinated, meaning that all countries of the coalition will play the same strategy. Because, $\mathcal{C}_{\text{PEP}}$ is also rational, it will play the best response strategy to the dominant strategy of $\mathcal{C}_{\text{VAC}}$. It means that, $\mathcal{C}_{\text{PEP}}$ will also play the vaccination strategy. We can see that a Nash equilibrium arises; this would be $\left(\text{vac}_{\mathcal{C}_{\text{VAC}}}, \text{vac}_{\mathcal{C}_{\text{PEP}}}\right)$.

Game analysis: non-cooperative repeated game setting

If we have no confidence that the coalition $\mathcal{C}_{\text{PEP}}$ is coordinated, and each country can play independently. We are, as in the beginning, in the non-cooperative setting. We considered that the game is played only once, but unless the rabies is eliminated, the game is bound to be repeated. Therefore, we can argue that if there is no cooperation between all players in the first year, after a certain amount of time, the game must be replayed when the pathogen is reintroduced everywhere. At the second round, as well as all consecutive rounds, to the projected payoff, we must add the payoff of the years between rounds. The payoff is always negative, so the total payoff function is strictly decreasing. We saw before that mass vaccination by all countries $\mathbf{s}_{\mathcal{C};\mathbf{VAC}}$ is Pareto-optimal, following the folk theorem[25], for an infinite repeated game, all Pareto-optimal strategies are Nash equilibriums. In our case, it is even more robust because the game stops if this strategy profile is played, so the best option is to play it in the first round. It is similar to Rubinstein's bargaining[26] model with discounting, except that we have a supergame instead of an extensive game.

Supplementary Information 3. The different layers of the game

Disperse evidence is available on how rabies elimination could be sustainably achieved. Still, implementation has yet to follow. We structure a holistic approach of rabies elimination in Africa according to Obrist's' multi-layered social resilience framework on four levels, taking the underlying competencies and threats into consideration[27].

The first level, the household-to-household level, is where an individual must decide whether to vaccinate one's dog. In Chad, only 25% of dog owners are willing to pay for the vaccination of their dog[28]. The willingness to pay (WTP) for rabies vaccination clearly depends on household income as in high income countries, the population readily accepts compulsory dog rabies vaccination. If the WTP is too low to achieve sufficient vaccination coverage as in Chad, then this situation corresponds to a public goods problem, a multiplayer prisoner dilemma. Empirical data shows that the perceived costs of vaccinating one's canine outweigh the perceived benefits. This utility perception can nonetheless be shifted by implementing awareness campaigns and providing the vaccination free of charge.

A **second level** is the district or province, reflecting administrative units that organize dog mass vaccination campaigns through veterinary authorities and/or the provision of PEP through public and private health providers. At the outset, the currently best situation is available PEP in public health facilities or private pharmacies. The patient or its caretaker, rarely the owner of the biting animal, pays for the PEP. PEP is often not available in sufficient quantities, especially not in remote rural areas. Many patients die because of the lack of available PEP: a tragedy of the absence of the public consensus to provide PEP, or the means to mass vaccinate dogs that would interrupt transmission[8, 28]. This is a coordination game between the public health and the veterinary authorities. The gains nonetheless get realized for the public health authorities through a cost that is borne by the veterinary authorities. This utility perception could be changed through transfer payments between the two. But even if rabies is successfully eliminated at the district or provincial level, by the nature of its transmission, it is reintroduced from the outside, requiring cooperation at the next level of social organization, the national state.

The national state is then the **third level** of cooperation which could lead to the successful elimination of rabies if sufficiently well-funded and organized properly[11]. This level has the same structure as the second level game, but instead the national states coordinate here. Similarly, even large nation-states are not protected from rabies reintroduction from

neighboring countries, requiring international coordination similar to the one in Latin America[29]. The elimination of rabies in Africa requires a similar coordination mechanism to avoid the introduction of rabies from areas where it has been eliminated[30].
Regional coordination is then the **fourth level** of cooperation, which could be coordinated by the African Union Inter-African Bureau for Animal Resources (AU-IBAR) and the organizations of economic cooperation. Theoretically, this situation resembles the global public good problem under the weakest link technology[31]. As examples for the weakest link, technology can be found ranging from building dykes[31] or curbing the spread of an epidemic[32]. The underlying idea here is that to provide the common public good, transfers targeted at the weakest link, e.g., the country with the smallest incentive to participate, will enable the participation of all countries and therefore enable the provision of the common public good. As we have shown through our analysis that even with pathogen reintroduction the OH approach is superior opposed to solely administering PEP. This opens additional financial leeway for transfer payments between countries, the organization of regional unions with border protection, or even costly border protection for single countries. Thus, providing additional tools to increase the incentives even further for regional collaboration.

References Supplementary Information

1. Laager M*, et al.* A metapopulation model of dog rabies transmission in N'Djamena, Chad. *J Theor Biol* **462**, 408-417 (2019).

2. Mindekem R*, et al.* Cost Description and Comparative Cost Efficiency of Post-Exposure Prophylaxis and Canine Mass Vaccination against Rabies in N'Djamena, Chad. *Front Vet Sci* **4**, 38 (2017).

3. Zinsstag J*, et al.* Transmission dynamics and economics of rabies control in dogs and humans in an African city. *Proceedings of the National Academy of Sciences of the United States of America* **106**, 14996–15001 (2009).

4. Knobel DL*, et al.* Re-evaluating the burden of rabies in Africa and Asia. *Bull World Health Organ* **83**, 360-368 (2005).

5. UN. World Populations Prospects 2019.). Departement of Economic and Social Affairs (2019).

6. Frey J*, et al.* Survey of animal bite injuries and their management for an estimate of human rabies deaths in N'Djaména, Chad. *Tropical Medicine & International Health* **18**, 1555-1562 (2013).

7. Shim E, Hampson K, Cleaveland S, Galvani AP. Evaluating the cost-effectiveness of rabies post-exposure prophylaxis: a case study in Tanzania. *Vaccine* **27**, 7167-7172 (2009).

8. Zinsstag J*, et al.* Vaccination of dogs in an African city interrupts rabies transmission and reduces human exposure. *Sci Transl Med* **9**, (2017).

9. Zinsstag J, Schelling, E., Waltner-Toews, D., Whittaker, M., Tanner, M. *One Health: The theory and practice of integrated health approaches*. CABI (2015).

10. Hindmarsh A. LSODE. Ordinary Differential Equation System Solver. (1983).

11. Anyiam F*, et al.* Cost-estimate and proposal for a development impact bond for canine rabies elimination by mass vaccination in Chad. *Acta Trop* **175**, 112-120 (2017).

12. World Bank. GDP per capita (current US$).). 16.12.2021 edn (2021).

13. IMF. GDP per capita, current prices.). October 2021 edn (2021).

14. Tetchi MS*, et al.* Risk factors for rabies in Côte d'Ivoire. *Acta Trop* **212**, 105711 (2020).

15. Hampson K*, et al.* Estimating the Global Burden of Endemic Canine Rabies. *PLOS Neglected Tropical Diseases* **9**, e0003709 (2015).

16. Cleaveland S, Fèvre EM, Kaare M, Coleman PG. Estimating human rabies mortality in the United Republic of Tanzania from dog bite injuries. *Bull World Health Organ* **80**, 304-310 (2002).

17. Mindekem R, Kayali U, Yemadji N, Ndoutamia AG, Zinsstag J. [Impact of canine demography on rabies transmission in N'djamena, Chad]. *Medecine tropicale : revue du Corps de sante colonial* **65**, 53-58 (2005).

18. Kayali U*, et al.* Incidence of canine rabies in N'Djamena, Chad. *Prevvetmed* **61**, 227-233 (2003).

19. OECD. Working age population (indicator).) (2021).

20. Johannesson M. The willingness to pay for health changes, the human-capital approach and the external costs. *Health Policy* **36**, 231-244 (1996).

21. Sobol IM. Sensitivity analysis for non-linear mathematical models. *Math Modeling Comput Exp* **1**, 407-414 (1993).

22. Saltelli A. Making best use of model evaluations to compute sensitivity indices. *Computer Physics Communications* **145**, 280-297 (2002).

23. Savage LJ. *The foundations of statistics*. Courier Corporation (1972).

24. Von Neumann J, Morgenstern O. Theory of games and economic behavior. In: *Theory of games and economic behavior*). Princeton university press (1944).

25. Friedman JW. A Non-cooperative Equilibrium for Supergames. *The Review of Economic Studies* **38**, 1-12 (1971).

26. Rubinstein A. Perfect Equilibrium in a Bargaining Model. *Econometrica* **50**, 97-109 (1982).

27. Obrist B, Pfeiffer C, Henley R. Multi-layered social resilience: a new approach in mitigation research. *Progress in Development Studies* **10**, 283-293 (2010).

28. Dürr S, Meltzer M, Mindekem R, Zinsstag J. Owner valuation of rabies vaccination in dogs, Chad. *Emerging Infectious Diseases* **14**, 1650-1652 (2008).

29. Belotto AJ. The Pan American Health Organization (PAHO) role in the control of rabies in Latin America. *Developments in biologicals* **119**, 213-216 (2004).

30. Helle C*, et al.* Rabies control and elimination in West and Central Africa. *Acta Trop* **226**, 106223 (2021).

31. Hirshleifer J. From Weakest-Link to Best-Shot: The Voluntary Provision of Public Goods. *Public Choice* **41**, 371-386 (1983).

32. Caparrós A, Finus M. Public good agreements under the weakest-link technology. *Journal of Public Economic Theory* **22**, 555-582 (2020).
