# Supplementary material for: Benefit-cost analysis of coordinated strategies for control of rabies in Africa
Source: Nat Commun. 2023 Sep 7;14:5370. doi: 10.1038/s41467-023-41110-2 (PMC10484917; doi:10.1038/s41467-023-41110-2)
Supplement: Supplementary file 3 — Description of Additional Supplementary Files [file 41467_2023_41110_MOESM3_ESM.pdf]

Description of Additional Supplementary Files

Supplementary Data 1: Data used for the simulations for every country

Supplementary Data 2: Strategy analysis for every country

Supplementary Data 3: Sobol indices

Supplementary Data 4: Neighboring countries matrix

Supplementary Data 5: Minimal distance matrix

Supplementary Data 6: GDP data for every country

Supplementary Data 7: Dog population data for every country

Supplementary Data 8: Human rabies disease burden data for every country

Supplementary Data 9: Expanded version of Table 2 with precise values of country-specific input data for SEIR model and additional information
